# On a Newly-discovered Characteristic of Albumen, with Particular Reference to the Possibility of Its Being Mistaken for Caseine

**Published:** 1846-06

**Authors:** J. Underwood

**Affiliations:** Colchester


*On a Newly-discovered Characteristic of Albumen, with particular reference to the possibility of its being mistaken for Caseine.* By J. Underwood, Esq., Colchester.—In Number 24, vol. ii, of the Lancet for 1845, (new series,) there is an article, by Dr. Ure, upon the potato malady, and to the following paragraph, extracted therefrom, I would beg to call particular attention, as it is very likely to mislead those who are not conversant with the late discoveries in organic chemistry.

"Liebig imagines the essence of the disease to consist in the conversion of the albumine, a usual constituent of healthy potatoes, into caseine—a principle which, by its great instability of composition, is supposed to cause the potato to putrefy rapidly. I have subjected this opinion to the test of experiment. Perfectly sound potatoes, as also diseased ones, were sliced or grated, and separately digested in a very dilute alkaline ley at a blood heat. The infusions, when cool, being filtered and faintly acidulated with dilute acetic acid, afforded respectively a like proportion of caseine-looking flakes. It would thus appear, from this mode of testing, as prescribed by Dumas, in the seventh volume of his 'Traité de Chimie,' that sound potatoes contain as much caseine as unsound."

But I suppose Dumas and Ure, both of them, forget that Mülder, of Amsterdam, some time back, discovered that when either albumen, caseine, or fibrine were dissolved in caustic alkali, these solutions precipitated, upon the addition of a little excess of acid, a substance in appearance identical with caseine, but differing chemically therefrom in being a compound simply of carbon, hydrogen, nitrogen, and oxygen, while caseine is a compound of carbon, hydrogen, nitrogen, oxygen, and sulphur. To this substance he gives the name of protein, supposing that it is the common fundamental principle of albumen, caseine, and fibrine, which it forms by uniting with certain small quantities of sulphur, or sulphur and phosphorus, and which latter bodies are separated by the alkali, and retained in solution. When the protein is precipitated by an acid, the precipitate obtained therefrom by Ure was not caseine, but the protein of Mülder; or we are justified in supposing this to be the case till Ure or Dumas shall have proved by ultimate analysis that it is not.

There is another test for albumen to which Ure doubtless did, or at any rate ought to have had recourse, though he does not tell us so—that is, the supposed property of all albuminous fluids to coagulate by heat; and were this characteristic universal, it would be a good test; but I find, that when once we have dissolved albumen in caustic alkali, we are enveloped in many difficulties, from which time, perseverance, and experiment only can liberate us. I trust, ere long, to be able to do this; but for the present, I would only state my results as far as they go. I find, that when thus dissolved in certain proportions, it entirely loses the property of coagulating by heat, and that in proportions where it does not lose this property entirely, it does so to a great extent. A quantity of albumen is left in solution after all has been coagulated that heat will render insoluble, and protein is precipitated from this solution upon the addition of a slight excess of acid.

I think, therefore, that until the various properties of albuminous fluids can be fixed by definite laws, the conflicting statements of Liebig and Ure must fall to the ground, and we must seek elsewhere for the grand fundamental cause of that change which has plunged many parts of our own, together with our sister country Ireland, into a state of destitution and want, than in the conversion of the albumen into caseine.

I trust that these remarks will throw the path open for other experiments, and that others will follow it.—*Ibid.*

